# Outbreak detection algorithms for seasonal disease data: a case study using ross river virus disease

**DOI:** 10.1186/1472-6947-10-74

**Published:** 2010-11-24

**Authors:** Anita M Pelecanos, Peter A Ryan, Michelle L Gatton

**Affiliations:** 1 Malaria Drug Resistance and Chemotherapy Laboratory, Queensland Institute of Medical Research, Brisbane, Australia; 2 Mosquito Control Laboratory, Queensland Institute of Medical Research, Brisbane, Australia

## Abstract

**Background:**

Detection of outbreaks is an important part of disease surveillance. Although many algorithms have been designed for detecting outbreaks, few have been specifically assessed against diseases that have distinct seasonal incidence patterns, such as those caused by vector-borne pathogens.

**Methods:**

We applied five previously reported outbreak detection algorithms to Ross River virus (RRV) disease data (1991-2007) for the four local government areas (LGAs) of Brisbane, Emerald, Redland and Townsville in Queensland, Australia. The methods used were the Early Aberration Reporting System (EARS) C1, C2 and C3 methods, negative binomial cusum (NBC), historical limits method (HLM), Poisson outbreak detection (POD) method and the purely temporal SaTScan analysis. Seasonally-adjusted variants of the NBC and SaTScan methods were developed. Some of the algorithms were applied using a range of parameter values, resulting in 17 variants of the five algorithms.

**Results:**

The 9,188 RRV disease notifications that occurred in the four selected regions over the study period showed marked seasonality, which adversely affected the performance of some of the outbreak detection algorithms. Most of the methods examined were able to detect the same major events. The exception was the seasonally-adjusted NBC methods that detected an excess of short signals. The NBC, POD and temporal SaTScan algorithms were the only methods that consistently had high true positive rates and low false positive and false negative rates across the four study areas. The timeliness of outbreak signals generated by each method was also compared but there was no consistency across outbreaks and LGAs.

**Conclusions:**

This study has highlighted several issues associated with applying outbreak detection algorithms to seasonal disease data. In lieu of a true gold standard, a quantitative comparison is difficult and caution should be taken when interpreting the true positives, false positives, sensitivity and specificity.

## Background

Disease surveillance and outbreak detection are fundamental to the provision of adequate and timely public health services. There are a multitude of outbreak detection algorithms that have been applied to a variety of disease studies at different spatial scales. The United Kingdom utilises a log-linear regression model via an nationwide automated system to detect abnormalities in the occurrence of infectious diseases [1]. Hidden Markov Models (HMMs) and Bayesian HMMs have been used for influenza epidemic detection [2] and hepatitis A disease surveillance respectively [3], while a compound smoothing technique has been applied to Salmonella and Shigella notification data in Australia [4]. Application of space-time scan statistics to hospital emergency department visits have been used to anticipate disease outbreaks [5]. Other types of outbreak algorithms include time series methods, mean-regression methods and autoregressive integrated moving average (ARIMA) models.

Although many detection algorithms have been reported, there are few studies comparing methods, especially using public health data. The extensively used Early Aberration Reporting System (EARS) C1, C2 and C3 algorithms have been assessed and compared using artificial simulations that mimic public health data [6-8] and semi-synthetic disease data [9]. The historical limits method (HLM) has also been assessed against the EARS C1, C2 and C3 methods using simulated data [6]. Watkins *et al*. [10] compared the sensitivity and timeliness of the EARS C1, C2 and C3 methods and a negative binomial cusum outbreak detection method to detect aberrations in Ross River virus (RRV) disease in Western Australia.

Mosquito-borne diseases such as malaria, dengue, West Nile virus, RRV disease and chikungunya have a strong seasonal pattern in most regions of the world. This seasonality potentially impacts on the utility of some outbreak detection methodologies, specifically when the application is to detect aberrations beyond the usual seasonal pattern, instead of detecting the start of the season. Here we apply a sub-set of five commonly used outbreak detection methodologies to seasonal disease data, using RRV disease as a case study, and compare the ability of the methods to detect outbreaks above the expected seasonal pattern in cases.

## Methods

### Notification and population data

RRV disease notification data from January 1991 to December 2007 was supplied by Queensland Health. Access to this data is restricted and is granted upon request on a case-by-case basis. The data from January to June 1991 were used as historical data only while reported data were from July 1991 to June 2007. In Queensland, Australia, serologically-confirmed RRV disease cases must be reported to Queensland Health, usually by the pathology testing laboratory. The notification data received for each de-identified patient included the onset week of illness, age (0-29, 30-59 and ≥60 years), gender and local government area (LGA) of residence. Notification data for patients residing in the LGAs of Brisbane, Emerald, Redland and Townsville were selected for this study due to their contrasting population sizes and disease incidence rates. Patient data were aggregated to represent total notifications by week of onset of illness and LGA.

Annual population data for each LGA was obtained from the Australian Bureau of Statistics [11]. Populations were categorised by age and gender to match the categories used in the notification data.

### Defined signal period

To have a reference point to compare the methods, we established a defined signalling period (DSP). The average number of notifications for each week was calculated and the difference between the actual notifications and the average for each week was determined. Because the peak RRV activity does not occur at exactly the same time every year, we allowed the annual notification data to be shifted by a maximum of 2 weeks in either direction and recalculated the difference between the average and shifted data. The optimal shift for each year's data was determined by minimising the sum of squares for the difference between the weekly notifications and the average number of notifications for the same week, using only data from weeks 51 to 27 (peak transmission season) in the sums of squares calculation. The data for the difference between the actual notifications (incorporating the optimal shift) and the average notifications formed the basis for defining a DSP. A DSP occurred if there were 4 or more consecutive positive values (positive difference between the shifted actual and average notifications), continuing until a negative value was encountered. Preliminary investigation revealed that allowing the data to be shifted +/-2 weeks did not affect the number of DSP periods identified, instead impacting only on the timing of the DSP. Incorporation of data shifting sometimes resulted in the DSP commencing 1-3 weeks earlier than corresponding DSPs where no data shifting procedure was applied.

Algorithms were individually compared to the DSP to determine their ability to detect outbreaks. Each outbreak signal was classified as a true positive (TP) or a false positive (FP). To classify as a TP, the outbreak signal from the algorithm had to overlap with a DSP. If the signal did not overlap with a DSP it was categorised as a FP. The percentage of DSPs not detected by each algorithm, the false negative (FN) rate, was determined across the entire year and also during the main transmission season (1 December - 30 April; Peak FN).

### Outbreak detection algorithms

Five different types of outbreak detection algorithms were investigated, and 17 algorithm variants were applied prospectively. The algorithms fell into two broad categories; those that used historical data and those that did not. Each method was independently applied to the data for each of the four LGAs. A summary of the parameters used is contained in Additional file 1 Table S1.

#### EARS algorithm

The EARS algorithms applied in this analysis were calculated as previously reported [7]. Using the data from the Brisbane LGA, we explored the effect of altering *k*, an arbitrary constant chosen to explain the variation of the mean of the baseline period. For all other LGAs we used *k *= 3 since this value appeared to adequately explain the variation of the baseline mean without inhibiting the identification of many true signals. An outbreak event was declared when the cumulative sum for a period exceeded the threshold value, *h*. In the absence of any previous data on the optimal value for *h*, we used values from 2 to 15. Since our data had a weekly interval, both the C2 and C3 algorithms were applied using a one week guard band (GB), that is, a 1 week gap between the baseline and the week of investigation. The C1 algorithm was applied with no GB (no gap between the baseline and week of investigation). Each of the EARS C1, C2 and C3 variants were applied with three different baseline periods; 2, 4 and 8 weeks. EARS algorithms with baselines temporally close to the current week of analysis are not largely influenced by seasonal effects [6], and thus were not adjusted for seasonality.

#### Negative binomial cusum (NBC)

The NBC method (originally proposed in Hawkins & Olwell [12]) was developed to reduce the number of false positives generated by other cusum methods when applied to over-dispersed data [10]. We applied this algorithm following the protocol of Watkins *et al*. [10] with the "out of control mean" set to 3 and signal threshold levels ranging from 2 to 15.

The NBC method does not account for seasonality and in lieu of published information about the impact of seasonality on the detection of outbreaks we conducted the analysis independently using both raw and seasonally adjusted data. The seasonally-adjusted variant of the algorithm used transformed notification data: $X_{i}^{*}=\frac{Y_{j,m}}{S_{j}}$, where *Y_j, m _*was the number of notifications in week *j *(*j *= 1,...,52) of year m (m = 1,...,17), *i *= 52(m-1)+*j*, and $S_{j}=\frac{\frac{1}{17}\Sigma_{m=1}^{17}Y_{j,m}}{\frac{1}{\text{SS}4}\Sigma_{m=1}^{17}\Sigma_{j=1}^{\text{S}2}Y_{j,m}}$. The standard NBC method was applied using the transformed data, with an out of control mean and outbreak threshold level of 2.

#### Historical limits method (HLM)

The HLM [13] incorporates historical data and accounts for seasonality by design, unlike the cusum methods. An outbreak signal occurs when:

$$\frac{x_{0}}{\mu }>1+\frac{2\sigma_{x}}{\mu }$$

where *x_0 _*is the number of reported cases in the current period and *μ *and *σ_x _*are the mean and standard deviation of the historical data. In this study the method has been applied using weekly data from a) three consecutive periods (the current week, the preceding week and the subsequent week) over 5 years of historical data (total of 15 data points), and b) from five consecutive periods (the current week plus 2 weeks either side) over 5 years (25 data points).

#### Poisson outbreak detection (POD) method

The POD method was applied using the procedure previously reported [14]. Since our dataset commenced in 1991, we started the analysis of outbreaks in 1996 using 5 years of baseline data. From this point the number of years of baseline data was increased year by year so that by 2001 the baseline data set contained 10 years of historic data. From 2001 onwards, the preceding 10 years of data was used as a baseline. We applied the method using 2-week and 4-week moving windows. An outbreak was declared when the number of notifications exceeded the 95^th ^percentile of the Poisson cumulative distribution for the current window.

#### Purely temporal SaTScan

SaTScan™ is used for conducting spatial, temporal and space-time analyses and is based on identifying maximum likelihood clusters for a scanning window that moves across space and/or time [15]. We used the software to conduct a Poisson purely temporal prospective analysis for each LGA to look for temporal windows with high incidence rates. The program was implemented using weekly time units, a p-value cut-off of 0.05 with a maximum temporal cluster size of 60 days. The prospective analysis was conducted using the same historic data as the POD method.

To adjust for seasonality within the SaTScan program, we scaled the population sizes. The factor used to scale the population sizes was dictated by the average incidence rate for a given week. This average incidence rate during the 17 year study period was calculated for each week of the year, as was the total average incidence (the average of the 52-weekly average incidence rates). The scaled populations used in SaTScan were then calculated as:

$${\text{population}}_{i}=\text{annual population}\times \left(\text{average incidence for week }i\right)/\left(\text{total average incidence}\right).$$

When the weekly average incidence was zero, the annual population was used since the choice of population was irrelevant. Scaling the weekly populations had the effect of increasing the population during the high transmission season, thereby drawing the incidence rate closer to the average baseline level.

## Results

Over the study period 35,019 notifications for RRV disease were reported for the state of Queensland. Of these notifications 9,188 (26.2%) were from people living within Brisbane, Redland, Emerald and Townsville LGAs. These four LGAs represented approximately 32% of the Queensland population. Brisbane had the largest number of notifications along with the largest population (Table 1).

**Table 1:** RRV disease notifications (July 1991 - December 2007) and population sizes for Brisbane, Emerald, Redland and Townsville, as well as for all of Queensland.

LGA	Total Notifications	Est. resident popn (1991)	Est. resident popn (2007)
Brisbane	5,189	769,087	992,176
Emerald	257	9,842	15,364
Redland	836	82,818	131,332
Townsville	2,906	86,245	102,020
Queensland	35,019	2,977,772	4,181,431

### Parameter selection

The EARS algorithms required 3 parameters to be specified: *h*, *k *and baseline period. To examine the responsiveness of the EARS C1, C2 and C3 algorithms to the values chosen for the baseline period and *k*, we applied each variant to the data from Brisbane. We used *h *= 2, as previously reported [10], a baseline period of 4 or 8 weeks and 3 ≤ *k *≤ 6 (Table 2). The frequency of miscellaneous signals lasting only 1 or 2 weeks was higher for all EARs algorithms using the shorter 4-week baseline period, compared to the 8-week baseline. Within each algorithm, the frequency and duration of signals tended to decrease as *k *increased (Table 2).

**Table 2:** Summary of signals generated by EARS C1, C2 and C3 cusum methods.

Method & parameters	No. signals of specified duration				Details of signals lasting ≥4 wks
		
	1 wk	2 wks	3 wks	≥4 wks	
C1 4 wk BL k = 3	5	6	1	5	1992 (wk 5-11), 1996 (3-12), 2003 (10-20), 2004 (4-14), 2007 (38-45)

C1 4 wk BL k = 4	5	4	0	5	1992 (wk 5-9), 1996 (5-9), 2003 (10-18), 2004 (4-7), 2007 (38-43)

C2 4 wk BL k = 4	7	5	2	5	1992 (wk 5-13), 1996 (5-14), 2003 (11-25), 2004 (4-17), 2006 (2-7)

C2 4 wk BL k = 5	7	1	1	4	1992 (wk 5-12), 1996 (5-13), 2003 (11-23), 2004 (4-15)

C2 4 wk BL k = 6	5	2	1	4	1992 (wk 5-11), 1996 (5-12), 2003 (11-21), 2004 (4-8)

C3 4 wk BL k = 5	8	1	2	4	1992 (wk 5-12), 1996 (5-12), 2003 (11-15), 2004 (4-15)

C1 8 wk BL k = 3	6	3	1	3	1992 (wk 5-13), 1996 (5-14), 2003 (11-19)

C1 8 wk BL k = 4	6	0	0	3	1992 (wk 5-10), 1996 (5-11), 2003 (11-15)

C2 8 wk BL k = 4	0	4	0	6	1992 (wk 5-15), 1996 (5-18), 2001 (13-16), 2003 (11-21), 2004 (11-14), 2006 (2-8)

C2 8 wk BL k = 5	1	4	0	3	1992 (wk 5-13), 1996 (5-16), 2003 (11-18)

C3 8 wk BL k = 4	0	4	0	6	1992 (wk 5-15), 1996 (5-18), 2001 (13-16), 2003 (11-21), 2004 (11-14), 2006 (2-8)

C3 8 wk BL k = 5	1	4	0	3	1992 (wk 5-13), 1996 (5-16), 2003 (11-18)

Parameter values applied were *h *= 2 with 4 or 8 weeks of baseline (BL) data and 3 ≤ *k *≤ 6.

We investigated how the alteration of *h *and *k *levels in the C2 algorithm affected the number of outbreak signals, as well as the timing for the start of the signal. The 4 week baseline was used. Altering the *h *values between 2 and 15 and *k *values between 1 and 5 made little difference to the number of short (≤3 week) signals detected (data not shown). The start date of the signal was not noticeably different, although *h *values greater than 7 tended to delay the start of a signal by one week, particularly when coupled with higher values of *k *(data not shown).

The NBC algorithm was adversely affected by the seasonality in the data. Using an out of control mean of 2, it produced signals at the start of each transmission season (data not shown). This problem was reduced by using an out of control mean of 3, however many seasonal signals were still produced particularly for *h *= 2 and *h *= 4 (data not shown). To overcome this we developed a seasonally-adjusted version of the algorithm. This adjusted algorithm failed to produce many alert signals using 4 weeks of baseline data, instead requiring a longer baseline period of 8 weeks.

### Comparison of methods

**Figure 1:**
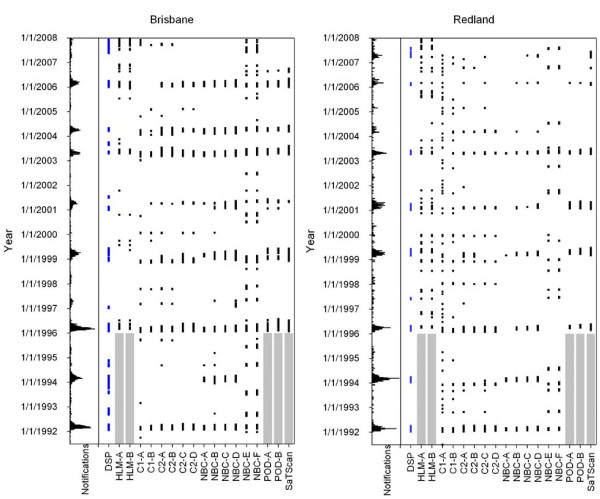
**Notifications, defined signal periods (DSP) and outbreak alerts for Brisbane and Redland LGAs**. Note that the scaling of the horizontal axes for number of notifications differs between subfigures. The grey shaded regions represent the years that no analysis could be performed due to the requirement of 5 years of historical data. HLM-A: HLM (15 wk BL); HLM-B: HLM (25 wk BL); C1-A: C1-4 wk BL (h = 2); C1-B: C1-8 wk BL (h = 2); C2-A: C2-4 wk BL (h = 4); C2-B: C2-4 wk BL (h = 6); C2-C: C2-8 wk BL (h = 4); C2-D: C2-8 wk BL (h = 6); NBC-A: NBC-4 wk BL no GB (h = 6); NBC-B: NBC-4 wk BL 1 wk GB (h = 6); NBC-C: NBC-8 wk BL no GB (h = 8); NBC-D: NBC-8 wk BL 1 wk GB (h = 8); NBC-E: Seasonally-adjusted NBC 8 wk BL 1 wk GB (h = 8); NBC-F: Seasonally-adjusted NBC 8 wk BL 2 wk GB (h = 8); POD-A: Poisson 2 wk Window; POD-B: Poisson 4 wk Window.

**Figure 2:**
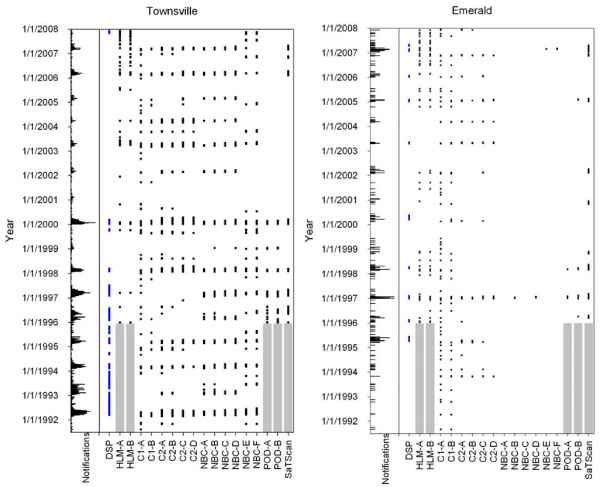
**Notifications, defined signal periods (DSP) and outbreak alerts for Townsville and Emerald LGAs**. Note that the scaling of the horizontal axes for number of notifications differs between subfigures. The grey shaded regions represent the years that no analysis could be performed due to the requirement of 5 years of historical data. Algorithm codes are as outline in Figure 1.

Given the preliminary analysis that centred on parameter selection for the Brisbane LGA described above, 17 variants of the 5 base algorithms were applied to the RRV data for Brisbane, Emerald, Redland and Townsville LGAs (Figure 1 and 2). The EARS C3 algorithm was not applied due to its similarity of results with the EARS C2 algorithm (Table 2). The EARS C1 and C2 and NBC algorithms were applied using *k *= 3.

Each of the methods examined was typically able to detect the same outbreaks. The exceptions were the seasonally-adjusted NBC methods that detected many short signals (Figure 1 and 2). The POD algorithm using a 4-week moving window returned less outbreak periods than its 2-week moving window counterpart, which tended to often have several segmented signals instead of one longer signal. There was little difference in the number of outbreaks detected by the HLM when 15 weeks of baseline data was used compared to 25 weeks of baseline data.

In this study, the DSPs were considered to be the benchmark against which the performance of algorithms was compared (Table 3 and 4). Overall, the largest outbreaks tended to be detected by most of the methods (Figure 1 and 2). In Emerald, the LGA with the smallest population and number of notifications, all of the methods had high FN rates ranging from 45% to 100% (Table 4). In larger LGAs the peak FN rate was usually lower than the FN over the entire year (Table 3 and 4). The NBC, POD and temporal SaTScan algorithms were the only methods that had TP rates >70%, FP (≥2 weeks) rates <20% and peak FN rates <20% (Table 3 and 4), although they did not obtain these results across every LGA.

**Table 3:** Summary of performance of outbreak detection algorithms for RRV data from Brisbane and Townsville.

Method	LGA							
	
	Brisbane				Townsville			
	
	TP	FP	FN (n = 19)	Peak FN (n = 9)	TP	FP	FN (n = 23)	Peak FN (n = 11)
HLM-A	40.0	10.0	54.5	62.5	12.9	19.4	50.0	60.0
			(n = 11)	(n = 8)			(n = 8)	(n = 5)
HLM-B	42.1	5.3	54.5	50.0	20.0	24.0	50.0	60.0
			(n = 11)	(n = 8)			(n = 8)	(n = 5)
C1-A	41.2	41.2	68.4	44.4	23.3	37.2	56.5	36.4
C1-B	46.2	30.8	68.4	44.4	35.0	45.0	69.6	45.5
C2-A	53.3	33.3	57.9	33.3	36.4	54.6	65.2	36.4
C2-B	58.3	16.7	63.2	22.2	31.6	36.8	73.9	45.5
C2-C	60.0	40.0	63.2	22.2	27.8	44.4	73.9	45.5
C2-D	60.0	30.0	68.4	33.3	41.7	33.3	78.3	54.5
NBC-A	81.8	9.1	52.6	22.2	57.1	19.1	56.5	18.1
NBC-B	73.3	13.3	42.1	11.1	57.9	31.6	56.5	18.1
NBC-C	88.9	11.1	52.6	0.0	57.1	42.9	60.9	18.1
NBC-D	72.7	27.3	47.4	33.3	53.3	40.0	60.9	18.1
NBC-E	40.6	34.4	47.4	33.3	44.8	13.8	47.8	36.4
NBC-F	40.6	37.5	47.4	33.3	46.9	21.9	39.1	27.3
POD-A	61.5	23.1	36.4	12.5	69.2	23.1	37.5	0.0
			(n = 11)	(n = 8)			(n = 8)	(n = 5)
POD-B	70.0	20.0	36.4	12.5	75.0	25.0	37.5	0.0
			(n = 11)	(n = 8)			(n = 8)	(n = 5)
SaTScan	62.5	25.0	45.5	25.0	44.4	44.4	50.0	20.0
			(n = 11)	(n = 8)			(n = 8)	(n = 5)

True positive (TP) values represent the percentage of signals that overlapped with a DSP, false positives (FP) are categorised as the percentage of signals lasting ≥2 weeks which do not overlap with a DSP, false negatives (FN) are the percentage of all DSPs that were not detected by the algorithm, and peak FN is the percentage of DSPs starting between 1 December and 30 April that were not detected by the algorithm. Algorithm codes are the same as detailed in Figure 1.

**Table 4:** Summary of performance of outbreak detection algorithms for RRV data from Emerald and Redland.

Method	LGA							
	
	Emerald				Redland			
	
	TP	FP	FN (n = 11)	Peak FN (n = 11)	TP	FP	FN (n = 11)	Peak FN (n = 8)
HLM-A	14.3	11.4	60.0	60.0	29.7	2.7	0.0	0.0
			(n = 10)	(n = 10)			(n = 9)	(n = 6)
HLM-B	14.7	11.8	60.0	60.0	29.0	7.9	0.0	0.0
			(n = 10)	(n = 10)			(n = 9)	(n = 6)
C1-A	7.4	13.0	63.7	63.7	19.0	10.3	45.5	25.0
C1-B	18.2	12.1	54.5	54.5	23.1	19.2	54.5	37.5
C2-A	38.5	30.8	45.5	45.5	33.3	33.3	45.5	25.0
C2-B	50.0	0.0	63.7	63.7	50.0	16.7	54.5	37.5
C2-C	33.3	41.7	63.7	63.7	46.7	33.3	36.4	12.5
C2-D	50.0	0.0	72.7	72.7	44.4	22.2	63.6	50.0
NBC-A	0.0	0.0	100.0	100.0	100.0	0.0	63.6	50.0
NBC-B	100.0	0.0	90.9	90.9	87.5	0.0	36.4	12.5
NBC-C	0.0	0.0	100.0	100.0	100.0	0.0	27.3	0.0
NBC-D	100.0	0.0	90.9	90.9	88.9	0.0	27.3	0.0
NBC-E	100.0	0.0	90.9	90.9	20.0	55.0	63.6	75.0
NBC-F	100.0	0.0	90.9	90.9	23.8	61.9	54.5	62.5
POD-A	50.0	0.0	90.0	90.0	87.5	12.5	33.3	16.7
			(n = 10)	(n = 10)			(n = 9)	(n = 6)
POD-B	75.0	0.0	70.0	70.0	71.4	14.3	33.3	16.7
			(n = 10)	(n = 10)			(n = 9)	(n = 6)
SaTScan	44.4	55.6	50.0	50.0	85.7	14.3	22.2	0 (n = 6)
			(n = 10)	(n = 10)			(n = 9)	

Reported values are as per Table 3.

The timing of signals was compared between algorithms for the two largest outbreaks to occur in each LGA after 1995 (Table 5). Overall there was little consistency between the algorithms that first detected outbreaks. For instance, the HLM method was last to detect both outbreaks in Brisbane and the 1996 outbreak in Redland, but first to detect the 2001 outbreak in Redland. Similarly the C1 and C2 algorithms detected the 1996 Redland outbreak before the other algorithms, but failed to detect the 1997 outbreak in Townsville and the 2007 outbreak in Emerald.

**Table 5:** Timing of signals for the two largest outbreaks in each LGA after 1995.

Method	LGA							
	
	Brisbane		Townsville		Redland		Emerald	
	
	1996	1998/99	1997	2000	1996	2001	1997	2007
DSP	5-21	50-4	6-20	2-11	7-18	1-15	1-7	5-9
		9-24						
HLM-A	10	ND	12	2-6	14-17	4-8	1-5	6-9
	12-13							
	17-21							
HLM-B	10	20	12	2-6	14-17	4-8	1-5	6-9
	12					11		
	18-21							
C1-A	3-12	46-47	12	2-8	5-7	5	1-2	ND
		50			9-10			
					14			
C1-B	5-14	46-48	ND	2-9	5-11	5	1-5	ND
		50						
C2-A	5-16	47-3	ND	2-15	5-16	5-8	1-4	ND
		7-8						
C2-B	5-15	47-48	ND	2-15	5-7	5-7	1-3	ND
		50-51			9-15			
C2-C	5-21	47-4	ND	2-16	5-16	5-9	1-6	ND
		7-12						
C2-D	5-21	47-48	ND	2-16	5-15	5-8	1-6	ND
		50-3						
NBC-A	5-13	7	4	2-7	ND	ND	ND	ND
		9-12	6-12					
NBC-B	5-15	1-16	4-15	2-8	10-15	5-8	2-3	ND
NBC-C	5-16	1-17	6-16	2-9	10-16	8	ND	ND
NBC-D	5-17	51-19	6-17	2-11	9-18	6-14	3-5	ND
NBC-E	6-17	47-7	9-12	2-8	ND	ND	ND	9-10
NBC-F	6-20	47-8	9-15	2-9	ND	ND	ND	9-11
POD-A	6-22	1-4	4	2-8	13-18	5-9	2-6	ND
		7	6-10			11-12		
		14-21	12-13			14-15		
		23	15			17-18		
POD-B	7-24	1-7	4-15	2-9	14-20	5-15	1-8	ND
		14-23				17-18		
						20		
SaTScan	5-24	50-23	6-19	2-12	9-21	4-18	1-11	3-17

Table entries are the weeks where an outbreak signal occurred, with Week 1 starting 1 January each year. ND, none detected.

## Discussion

Detection of outbreaks is an important part of disease surveillance for public health. Algorithms for outbreak detection should ideally provide early declarations of true signals but have low numbers of false positives. One way to improve the false positive rates of algorithms is to increase the threshold limits. However, by raising the threshold, it generally takes longer to detect an outbreak [16].

Our results for the influence of the EARS methods parameter choice on outbreak detection using seasonal disease data reflect those previously reported [9,17]. Specifically, an increase in *k *was associated with a decrease in the frequency and duration of signals, and a longer baseline period produced fewer short (≤3 weeks) signals. Since an increase in *k *reflected the need to have larger deviations away from the baseline mean to trigger a signal, we found that use of a larger *k *made it more difficult to detect outbreaks with a slow amplification phase.

The underlying seasonality in the data appeared to be problematic for the NBC algorithm, particularly when using an out of control mean of 2. Normalising the data to remove the regular seasonality stopped the annual signals associated with the start of the main transmission season, but instead produced many short signals. An alternative approach for adjusting for seasonality may be needed for this algorithm.

The HLM algorithm was sensitive to the frequency and magnitude of outbreaks detected in the previous 5 years. RRV disease outbreaks in Brisbane tended to occur at 2-3 year intervals, with the largest outbreaks occurring in 1993 and 1996. Because the later part of the study period tended to have smaller and less frequent outbreaks, there was a lack of consistency in the signals produced by the HLM algorithm, relative to the number of notifications in each outbreak. This issue was also apparent in Townsville.

The primary aim of this study was to investigate the performance of outbreak detection algorithms applied to seasonal data. Comparing the results was problematic, since this required a definition of a true outbreak or a gold standard. To overcome the subjective nature associated with visually identifying outbreaks, we defined the DSP and used it to determine the percent of signals that were TP, FP and FN. It should be noted that the DSP definition was arbitrary, and although it required four weeks or more of above average notifications, there was no minimum difference required between the actual and average notifications to define a signal. The four week criterion helped focus this study on outbreaks of public health importance for a relatively benign endemic mosquito-borne disease with no curative treatment or vaccine by disregarding outbreak signals of a short duration. This may not be appropriate for other seasonal diseases.

Most notifications of RRV disease occur during summer and autumn (December to April), imitating the dynamics of the mosquito vector. Therefore identification of the outbreaks during this period is a higher priority than for smaller outbreaks, which occur during the cooler months or at the end of the transmission season. In the three largest LGAs examined, the FN rate was generally lower when only outbreaks occurring between December and April were considered. It is likely that the TP and FP rates will follow a similar pattern, but this needs to be confirmed. Although we investigated the timeliness of each algorithm for the largest outbreaks in each LGA, there was no one method that consistently detected the outbreaks first. This may be due to differences in the characteristics of individual outbreaks such as rate of increase in notifications or the absolute number of notifications involved.

This study has highlighted several issues associated with applying outbreak detection algorithms to seasonal disease data. Outbreaks of significant size were identified by most of the algorithms applied. However some algorithms were prone to short, sporadic signals, particularly when applied to smaller populations with relatively few notifications. We also noted differences in the ability of the algorithms to detect outbreaks with a slower amplification stage compared to explosive outbreaks. This is a feature that may result in some methods working well on some disease data but not others. In lieu of a true gold standard, a quantitative comparison is problematical and caution should be used when interpreting TPs, FPs, sensitivity and specificity.

## Competing interests

The authors declare that they have no competing interests.

## Authors' contributions

AMP implemented the algorithms, analysed the results and drafted the manuscript.

PAR assisted with interpretation of the results and review of the manuscript.

MLG designed the study, assisted with reviewing the results and drafted the manuscript.

All authors read and approved the final manuscript.

## Pre-publication history

The pre-publication history for this paper can be accessed here:

http://www.biomedcentral.com/1472-6947/10/74/prepub

## Supplementary Material

Additional file 1 **Parameters used in each algorithm**. Summary description of the historical or baseline data used in each of the algorithms tested, along with information for the threshold values and guard bands used for the cusum algorithms. Click here for file
