# Supplementary material for: Outbreak detection algorithms for seasonal disease data: a case study using ross river virus disease
Source: BMC Med Inform Decis Mak. 2010 Nov 24;10:74. doi: 10.1186/1472-6947-10-74 (PMC3004813; doi:10.1186/1472-6947-10-74)
Supplement: Additional file 1 — Parameters used in each algorithm. Summary description of the historical or baseline data used in each of the algorithms tested, along with information for the threshold values and guard bands used for the cusum algorithms. [file 1472-6947-10-74-S1.PDF]

Supplemental Table 1. Parameters used in each algorithm. t refers to the week under investigation.

Algorithm	Historical/Baseline data	Threshold value (h)	Guard Band (GB) (wks)
HLM-A	(t-1, t, t+1) for preceding 5 yrs	-	-
HLM-B	(t-2, t-1, t, t+1, t+2) for preceding 5 yrs	-	-
C1-A	t-4, t-3, t-2, t-1	2	0
C1-B	t-8, t-7, t-6, t-5, t-4, t-3, t-2, t-1	2	0
C2-A	t-5, t-4, t-3, t-2	4	1
C2-B	t-7, t-6, t-5, t-4, t-3, t-2	6	1
C2-C	t-9, t-8, t-7, t-6, t-5, t-4, t-3, t-2	4	1
C2-D	t-9, t-8, t-7, t-6, t-5, t-4, t-3, t-2	6	1
NBC-A	t-4, t-3, t-2, t-1	6	0
NBC-B	t-5, t-4, t-3, t-2	6	1
NBC-C	t-8, t-7, t-6, t-5, t-4, t-3, t-2, t-1	8	0
NBC-D	t-9, t-8, t-7, t-6, t-5, t-4, t-3, t-2	8	1
NBC-E	t-9, t-8, t-7, t-6, t-5, t-4, t-3, t-2 (seasonally adjusted)	8	1
NBC-F	t-10, t-9, t-8, t-7, t-6, t-5, t-4, t-3 (seasonally adjusted)	8	2
POD-A	t for preceding 10 yrs*	-	-
POD-B	t for preceding 10 yrs*	-	-
Temporal SaTScan	Preceding 10 yrs*	-	-

* From 1996 to 2001 the amount of historic data increased from 5 to 10 years
